# The GOFURTGO Study: AGITG Phase II Study of fixed dose rate gemcitabine–oxaliplatin integrated with concomitant 5FU and 3-D conformal radiotherapy for the treatment of localised pancreatic cancer

**DOI:** 10.1038/bjc.2011.526

**Published:** 2011-12-01

**Authors:** D Goldstein, N Spry, M M Cummins, C Brown, G A van Hazel, S Carroll, S Selva-Nayagam, M Borg, S P Ackland, C Wratten, J Shapiro, I W T Porter, G Hruby, L Horvath, S Bydder, C Underhill, J Harvey, V J Gebski

**Affiliations:** 1 Department of Medical Oncology, Prince of Wales Hospital, High Street, Randwick, New South Wales 2031, Australia; 2 Faculty of Medicine, University of New South Wales, Kensington, New South Wales 2052, Australia; 3 Sir Charles Gairdner Hospital, Hospital Road, Nedlands, Western Australia 6009, Australia; 4 Faculty of Medicine, University of Western Australia, Crawley, Western Australia 6009, Australia; 5 NHMRC Clinical Trials Centre, 94-96 Parramatta Road, Camperdown, New South Wales 2050, Australia; 6 Royal Adelaide Hospital, North Terrace, Adelaide, South Australia 5000, Australia; 7 Calvary Mater Hospital, Edith Street, Waratah, New South Wales 2298, Australia; 8 Cabrini Medical Centre, 181-183 Wattletree Road, Malvern, Victoria 3144, Australia; 9 William Buckland Radiotherapy Centre, The Alfred Hospital, Commercial Road, Prahran, Victoria 3181, Australia; 10 Royal Prince Alfred Hospital, Missenden Road, Camperdown, New South Wales 2050, Australia; 11 School of Surgery, University of Western Australia, Crawley, Western Australia 6009, Australia; 12 Border Medical Oncology, Murray Valley Private Hospital, Wodonga, Victoria 3690, Australia; 13 Princess Alexandra Hospital, Woolloongabba, Queensland 4102, Australia

**Keywords:** chemoradiotherapy, conformal radiotherapy, locally advanced, pancreas cancer, unresectable

## Abstract

**Background:**

Locally advanced inoperable pancreatic cancer (LAPC) has a poor prognosis. By increasing intensity of systemic therapy combined with an established safe chemoradiation technique, our intention was to enhance the outcomes of LAPC. In preparation for phase III evaluation, the feasibility and efficacy of our candidate regimen gemcitabine–oxaliplatin chemotherapy with sandwich 5-fluorouracil (5FU) and three-dimensional conformal radiotherapy (3DCRT) needs to be established.

**Methods:**

A total of 48 patients with inoperable LAPC without metastases were given gemcitabine (1000 mg m^−2^ d1 + d15 q28) and oxaliplatin (100 mg m^−2^ d2 + d16 q28) in induction (one cycle) and consolidation (three cycles), and 5FU 200 mg m^−2^ per day over 6 weeks during 3DCRT 54 Gy.

**Results:**

Median duration of sustained local control (LC) was 15.8 months, progression-free survival (PFS) was 11.0 months, and overall survival was 15.7 months. Survival rates for 1, 2, and 3 years were 70.2%, 21.3%, and 12.8%, respectively. Global quality of life did not significantly decline from baseline during treatment, which was associated with modest treatment-related toxicity.

**Conclusion:**

Fixed-dose gemcitabine and oxaliplatin, combined with an effective and safe regimen of 5FU and 3DCRT radiotherapy, was feasible and reasonably tolerated. The observed improved duration of LC and PFS with more intensive therapy over previous trials may be due to patient selection, but suggest that further evaluation in phase III trials is warranted.

Pancreatic cancer has a 5-year survival rate of less than 5% (Jemal *et al*, 2005). Surgical resection offers the only chance of cure, but most patients present with inoperable metastatic or locally advanced disease (Cardenes *et al*, 2006). Chemotherapy alone has been shown to improve survival of locally advanced pancreatic cancer (LAPC) patients (7.4–13.0 months, median 9.8) (Hazel *et al*, 1981; Klaassen *et al*, 1985; GITSG, 1988; Berlin *et al*, 2002; El-Rayes *et al*, 2003; Rocha Lima *et al*, 2004; Louvet *et al*, 2005; Huguet *et al*, 2007; Chauffert *et al*, 2008; Loehrer *et al*, 2008; Poplin *et al*, 2009).

Combined chemotherapy and radiotherapy regimens without an induction, consolidation or maintenance phase have modest survival improvements (7.8–9.6 months, median 8.4) (Earle *et al*, 1994; Cohen *et al*, 2005; Wilkowski *et al*, 2009), and regimens with a consolidation or maintenance phase have performed better (6.7–14.5 months, median 9.5) (Hazel *et al*, 1981; Moertel *et al*, 1981; GITSG, 1985, 1988; Klaassen *et al*, 1985; Shinchi *et al*, 2002; Li *et al*, 2003; Chung *et al*, 2004; Wilkowski *et al*, 2006, 2009; Chauffert *et al*, 2008; Loehrer *et al*, 2008). However, the consistently best survival rates for inoperable locally advanced disease are consistently seen across multiple phase II studies that include neo/adjuvant chemotherapy components with various specific three-dimensional conformal radiotherapy (3DCRT) radiation techniques (11.7–15.0 months, median 12.9; Goldstein *et al*, 2007; Huguet *et al*, 2007; Ko *et al*, 2007; Moureau-Zabotto *et al*, 2008). These outcomes contrast with those reported by the only currently reported phase III study assessing the role of CRT (Chauffert *et al*, 2008). The investigators employed an unpiloted and novel ‘aggressive CRT technique’, which led to a substantially worse median survival at 8.6 months, suggesting that the choice of specific radiation technique is likely to be critical to successfully establish the role of combined modality therapy.

It is important to recognise that the anatomical position of the pancreas presents a difficult radiation-planning problem for the Radiation Oncologist, because of close proximity of key tolerance organs and potential risk of major treatment toxicity. These constraints have, in large part, been responsible historically for the very low use of radiotherapy in the management of this cancer (Osborne *et al*, 2006).

We established the safety and efficacy of our CRT regimen previously. Our earlier study of gemcitabine with 5-fluorouracil (5FU) and 3DCRT for LAPC achieved median overall survival (OS) of 11.7 months, median progression-free survival (PFS) of 7.1 months, and median time to failure of local control (LC) of 11.9 months (Goldstein *et al*, 2007; Spry *et al*, 2008).

However, combined gemcitabine–oxaliplatin has achieved a higher response rate (RR) than gemcitabine alone in recent studies, and in one randomised study, improved PFS but not OS; but in a subsequent study, this was not replicated (Louvet *et al*, 2005; Poplin *et al*, 2009). Notably, in the positive study, 30% of patients in each arm had locally advanced disease (Louvet *et al*, 2005). In contrast, the negative study had only 9% in each arm (Poplin *et al*, 2009). It was therefore postulated that adding oxaliplatin to our previous chemoradiation programme for LAPC might benefit LC, delay systemic spread and improve survival.

The GOFURTGO Study evaluated both feasibility and efficacy of an enhanced chemotherapy regimen of induction gemcitabine–oxaliplatin, then 5FU–3DCRT, followed by consolidation gemcitabine–oxaliplatin in patients with LAPC, to identify its potential candidacy for subsequent phase III evaluation.

## Materials and methods

The GOFURTGO Study was an investigator-initiated, open-label, multicentre, phase II, single-arm study in Australia, sponsored by the Australasian Gastro-Intestinal Trials Group (AGITG).

### Role of the funding source

Partial funding was provided by Sanofi-Aventis as an unrestricted grant, but there was no input into the conduct or analysis of the study by them. Study coordination and all statistical analyses were undertaken by the National Health and Medical Research Council Clinical Trials Centre (CTC), University of Sydney. AGITG members and CTC staff prepared the manuscript and reviewed all data independently.

### Patient eligibility criteria

Eligible patients were aged ⩾18 years, with a histological or cytological diagnosis of locally advanced pancreatic adenocarcinoma of the head or body of the pancreas, deemed inoperable by their surgeon. Locoregional disease was confirmed by dual-phase computed tomography (CT), and distant metastases were excluded by CT. Disease was required to be measurable according to Response Evaluation Criteria in Solid Tumours (RECIST), and inoperable according to surgical review. Other inclusion criteria included ECOG performance status (PS) of 0–2, and adequate bone marrow and renal function.

Exclusion criteria included prior cytotoxic chemotherapy, significant loss of body weight (>15% weight loss since diagnosis), and previous abdominal radiotherapy.

The study was conducted in accordance with the International Committee on Harmonisation Good Clinical Practice guidelines. Institutional ethics approval was obtained, and all patients provided written informed consent.

### Treatment regimen

**Figure 1:**
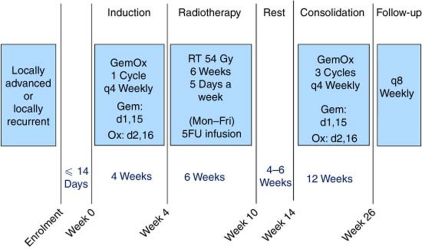
Study design.

Treatment consisted of one cycle of induction gemcitabine–oxaliplatin, followed by 6 weeks of radiotherapy with concurrent 5FU, 4–6 weeks of rest, and then three cycles of consolidation gemcitabine–oxaliplatin within 14 weeks (Figure 1).

Each cycle consisted of gemcitabine (1000 mg m^−2^ intravenous infusion over 100 min) on days 1 and 15, and oxaliplatin (100 mg m^−2^ intravenous infusion over 120 min) on days 2 and 16, of each 28-day cycle. During chemoradiotherapy, 5FU (200 mg m^−2^ per day) by continuous infusion began on the first day of radiotherapy and continued until completion of radiotherapy. Dose-modification criteria were defined in the protocol. After study completion, further treatment was administered at the discretion of the treating physician.

Radiotherapy was to start within 6 weeks of the commencement of induction chemotherapy and consisted of 54 Gy in 30 daily fractions of 1.8 Gy, as previously reported (Goldstein *et al*, 2007). Radiotherapy quality assurance was as previously described (Spry *et al*, 2008), with the addition of pretreatment assessment of each patient's radiotherapy plan, using a radiotherapy quality-assurance planning programme (SWAN; Ebert *et al*, 2008). This ensured that all plans for treatment met the protocol specifications. Further details are shown in the Supplementary Information.

Treatment was permanently discontinued in the event of progressive disease, patient or physician request, or excessive toxicity.

### Patient evaluation

Before registration, each patient was assessed by complete physical examination, haematology, biochemistry, carbohydrate antigen (CA)19-9, and contrast-enhanced CT scans of the thorax, abdomen, and the pelvis. Haematology and biochemistry were repeated on day 1 and days 12–15 of every chemotherapy cycle, and weekly during chemoradiotherapy. CA19-9 was assessed before and after radiotherapy, at completion of treatment, and at every follow-up assessment.

A contrast-enhanced CT scan of the thorax, abdomen, and the pelvis was performed before radiotherapy began, before consolidation chemotherapy began, at completion of treatment, and then as clinically indicated. After distant progression, scanning continued for assessing LC. Patients were followed up every 2 months until death.

Quality of life (QOL) was assessed with the European Organisation for Research and Treatment of Cancer (EORTC) QOL Questionnaire C30, Version 2.0 (1995; Aaronson *et al*, 1993), pancreas-specific module (PAN26; Fitzsimmons *et al*, 1999), patient disease and treatment assessment (DATA) forms, and study-specific scales (Stockler *et al*, 2007). Patients completed questionnaires at baseline, before and after radiotherapy, before commencing consolidation chemotherapy, at completion of treatment, and at every follow-up assessment until disease progression. Change to the EORTC global domain was our *a priori* selected primary QOL endpoint. Scores at each assessment time were compared with the baseline score, to infer change according to treatment phase. Change between phases was calculated and classified as stable, improved or worsened using a 10-point cut-off to summarise QOL change over time.

### Statistical design and analysis

The primary endpoint of the study was feasibility, as assessed by the proportion of patients starting and finishing >80% of the planned dose on time for each component of treatment. The target enrolment of 45 patients was designed to ensure the 95% confidence interval (CI), for the primary endpoint (a proportion) would have a width of at most ±15%. Our goal was to establish if this approach was feasible in this challenging patient population in a single-arm smaller study and secondarily identify any indication to justify a larger sample size for a randomised phase II or definitive phase III trial. All SAEs were monitored by the Radiation and Medical Oncology principle investigators in real-time and a monthly summary sent to all members of the Trial Management Committee. At the 1-year time point, all SAEs were reviewed and it was concluded that the trial should continue. We chose to have a single-step continuous accrual with sufficient numbers to indicate viability of further exploration of this regimen. In addition to feasibility, any decision to proceed would need to be based upon a combination of factors including RR, time to progression, toxicity, treatment tolerance/QOL derived from the trial, that is, no single cut-point would suffice.

A detailed review of the definitions of response and time to progression are in the Supplementary Information.

Secondary endpoints included treatment-related toxicity, tumour RR, and time to failure of sustained LC, PFS, OS, CA19-9 RR, and QOL. OS is defined as the time from date of registration to death (or date of last follow-up). Progression-free survival is defined as time from registration to documented evidence of disease progression, the occurrence of new disease, or death from any cause.

As most previous studies ceased to monitor LC once systemic progression occurred, we assessed this as a separate outcome. Failure of sustained LC was defined as disease progression involving the pancreas, according to RECIST. Patients without a date of local progression were censored on the date of their last scan, except one who was censored on the date of definitive surgery.

Toxicity was graded according to the NCI Common Terminology Criteria for Adverse Events, version 3.0 (National Cancer Institute, Bethesda, MD, USA). RR were assessed in accordance with RECIST, version 1.0. Confirmation of response by consecutive CT scans was required. A best response of stable disease (SD) required at least two determinations of SD before disease progression.

Local control, PFS, and OS were described with the Kaplan–Meier curves measured from registration. Medians are reported with 95% CIs. CA19-9 response was defined in the protocol as the proportion of patients with a >50% reduction sustained for at least 6 weeks as per the Rustin criteria for CA125 (Rustin *et al*, 1996); however, as only four patients had two pretreatment samples, an exploratory analysis was performed instead, using data from all patients who had given at least one pretreatment sample (see Supplementary Information).

Exploratory analyses adjusting for age, disease stage, ECOG PS, CA19-9, and white cell count for PFS and OS were performed in a Cox regression model (see Supplementary Information). The impact of CA19.9 changes during the first 6 weeks of treatment was assessed using a landmark analysis (Anderson *et al*, 1983), using incidence of reduction in CA19.9 within the first 6 weeks as a predictor of subsequent PFS/OS. Reductions in CA19.9 of 50% were compared with RECIST response in a contingency table.

All analyses were by intention to treat, except radiation toxicity, which included only patients who had commenced radiotherapy. All *P*-values and 95% CIs were two-tailed, without any adjustment for multiple comparisons. Analyses used SAS (version 9.2, SAS Institute Inc., Cary, NC, USA), SPSS (version 18, IBM, New York, NY, USA) and ACCoRD (Analysis of censored and correlated data, Boffin Software, Eastwood, NSW, Australia).

### Comparison with previous study

Our previous study tested an identical treatment regimen without oxaliplatin (i.e., gemcitabine with sandwich 5FU–3DCRT; Goldstein *et al*, 2007). To allow results of the current and previous studies to be compared and to establish potential enhancement of efficacy, patient and study characteristics were compared (Table 4).

## Results

### Patient population

**Figure 2:**
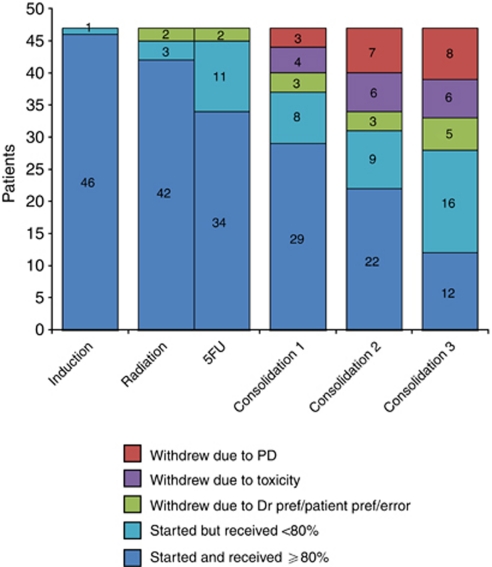
Patient disposition. Patients who withdrew from the study during a component are counted as started or completed <80% for that cycle, and counted according to reason for their withdrawal in subsequent components. Bar represents patients who completed all four components or reasons for early withdrawal. One out of seventeen patients who received ⩾80% of treatment did not complete according to specified time schedule.

Of 48 patients registered from 10 institutions between July 2005 and December 2007, one was ineligible because of the extent of disease (Figure 2). Baseline characteristics are shown in Table 1. Survival data closeout was 14 December 2009, providing median follow-up of 44.0 months for alive patients.

### Feasibility

Of 47 patients, 24 completed all planned cycles, and 17 of those patients (36% of the whole group) received >80% of the intended doses of all treatment. Median dose intensities compared with starting dosages were as follows: during induction, gemcitabine 100% and oxaliplatin 100% during chemoradiation, 5FU 98% in consolidation cycle 1, gemcitabine 100% and oxaliplatin 100% in consolidation cycle 2, gemcitabine 97% and oxaliplatin, 99% and in consolidation cycle 3, gemcitabine 76% and oxaliplatin 90%.

The planned radiation programme (Figure 2) appeared feasible. Of the 47 patients, 45 (96%) commenced chemoradiation, with 42 (93%) receiving >80% of all-planned radiation and 34 (76%) receiving >80% of 5FU.

Disease progression led to premature withdrawal of one patient after 11 Gy, and two withdrew because of toxicity at 36 and 43 Gy. Another three patients had minor dose reductions, because of toxicity and preference.

In all, 23 patients (49%) ceased treatment prematurely, 17% for toxicity, 19% for progressive disease, 4% for doctor preference, 2% for patient preference, and 6% for major protocol deviation by clinician (incorrect call of disease progression and incorrect number of consolidation chemotherapy cycles).

### Toxicity

**Figure 3:**
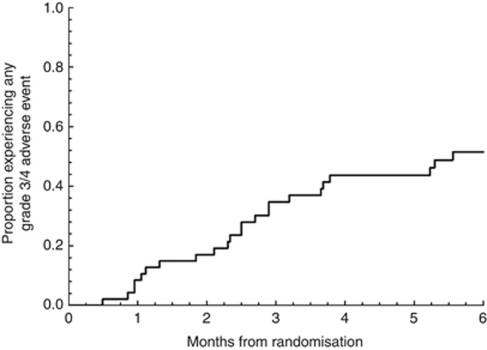
Cumulative incidence of any grade 3/4 toxicity (*n*=47).

The most common grade 3 events were anaemia, fatigue, nausea, diarrhoea, vomiting, neutropenia, infection, stomatitis, and anorexia (Table 2). The most common grade 4 toxicities were thrombocytopenia and liver function abnormalities. There were two late chemoradiation toxicity events: one patient with grade 3 and another with grade 4 gastric bleeding. Mortality from any cause at 60 days was 0%, and there were no treatment-related deaths. Cumulative incidence of any grade 3/4 toxicity is shown in Figure 3.

Four patients discontinued because of grade 2 or 3 toxicity during chemoradiation (fatigue; weight loss, nausea, and anorexia; abnormal liver function; weight loss, low albumin, and hypocalcaemia). Two discontinued treatment during the first consolidation chemotherapy cycle (abnormal liver function; anorexia), and a further two during the final cycle (allergic reaction; abnormal liver function, nausea, and vomiting).

### Efficacy

One patient was not evaluable for RR because of an obstruction requiring surgery after 2 days of treatment, who did not recover sufficiently to restart treatment, and did not have study assessments. Response rates are shown in Table 3.

Median time to loss of LC was 15.8 months (10.5–17.9), median PFS was 11.0 months (8.4–13.0), and median OS was 15.7 months (13.1–18.3). At the time of analysis, four patients were alive, one had ongoing LC and all had RECIST progression. Survival rates for 1, 2, and 3 years were 70.2%, 21.3%, and 12.8%, respectively.

Exploratory univariate analysis of age, disease stage, baseline PS, baseline CA19-9, and baseline white cell count were found to be not statistically significant predictors of PFS or treatment completion.

### Carbohydrate antigen 19-9

Twelve patients (26%) had a confirmed CA19-9 reduction of 50% or more. Exploratory landmark analysis of CA19-9 showed a trend towards shorter PFS for patients with higher CA19-9 levels 6 weeks after commencing treatment (HR 1.8; 95% CI 0.94–3.71; *P*=0.08). CA-19 reductions of ⩾50% were associated with longer PFS (9.5–11.5 months; HR 0.82; 95% CI 0.32–2.11; *P*=0.7); however, the size of this small subgroup limits the interpretation. CA19-9 reductions of ⩾50% were associated with RECIST response (OR 5.2; 95% CI 1.3–24; *P*=0.02). And 50% of the patients who had a 50% reduction in CA19-9 also had a confirmed response by RECIST, compared with 15% in those who did not.

### Quality of life

Fourty-five patients completed QOL questionnaires at baseline and were included in the global QOL analysis. Scores at each assessment time were compared with the baseline score, to infer change according to treatment phase. None of the changes compared with baseline were statistically significant for any treatment phase, a finding discordant with clinical experience. Osoba (2002) reported that a change of 10 points or more in a QOL measure is clinically important. We therefore investigated the relative proportions over time, showing a change higher than 10 points from baseline. At each of the five assessment times, approximately half (48–63%, median 54%) were unchanged, and the relative proportions improving (13–25%, median 17%) or worsening (21–39%, median 29%) did not greatly vary. An interesting pattern of change was seen when we analysed the change from one phase to the next. After the induction chemotherapy, 32% worsened and 14% improved. Across the chemoradiotherapy phase, 4% worsened and 17% improved. Across the consolidation chemotherapy phase, 58% worsened and only 8% improved. Chemotherapy phases appeared to affect global QOL in some patients, but scores returned to baseline in those completing all therapy (32 assessable of 34 patients). Analysis of QOL is the subject of another manuscript.

### Subsequent therapy

Salvage therapy was at the clinician's discretion and included further chemotherapy in 44% of cases. One patient had downstaging sufficient to have a Whipple's pancreaticoduodenectomy. After an R0 resection, the patient remained disease-free at final follow-up 20 months later.

### Comparison with previous study

**Figure 4:**
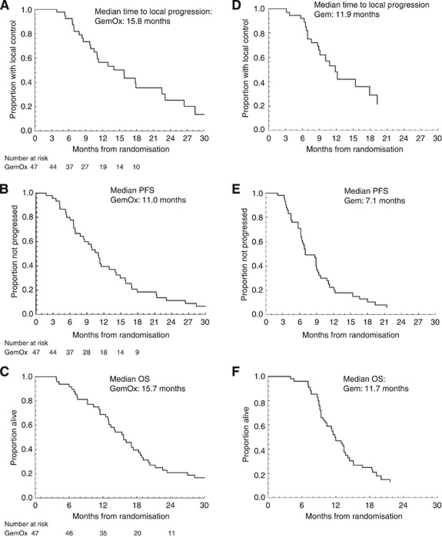
Kaplan–Meier curves of (**A**) sustained local control, (**B**) time to progression, and (**C**) overall survival in the current study of GemOx with 5FU–3DCRT (*n*=47); and (**D**) sustained local control, (**E**) time to progression, and (**F**) overall survival from our previous study of Gem with 5FU–3DCRT (*n*=41).

Although comparison of the two studies may be confounded due to differences in patient selection, most known prognostic confounders favoured the gemcitabine cohort (Table 4). Compared with our previous study using gemcitabine and the same radiation schedule, addition of oxaliplatin appeared to be associated with improved LC, PFS, and OS (previously 11.9 (8.9–17.9), 7.1 (6.3–9.2), and 11.7 (9.7–13.7) months, respectively, now 15.8 (10.5–17.9), 11.0 (8.4–13.0), and 15.7 (13.1–18.3) months, respectively; Figure 4; Table 5) without significantly increased toxicity.

## Discussion

This study has three important findings. This gemcitabine–oxaliplatin with sandwich 5FU–3DCRT regimen was associated with longer OS and LC than observed in our previous monotherapy programme, which warrants further evaluation in controlled trials. Second, it is a feasible and safe treatment programme. Finally, CA19-9 response 6 weeks after treatment commencement may be prognostic for survival.

In a cohort representative of community practice in terms of age and performance status, a median LC of 15.8 months and OS of 15.7 months compare favourably with other reports of regimens including chemotherapy before or after chemoradiation. In a recent review of trials including induction or maintenance chemotherapy, OS varied between 9.7 and 13.5 months, and LC varied between 7.1 and 10.5 months (Huguet *et al*, 2009). Our data suggest the need for better systemic therapy, but also the potential for optimised LC to influence outcomes in this subset of patients with LAPC. In particular, this approach may provide additional palliation by reducing intractable neuropathic pain, local bleeding, and gastric outlet obstruction, all of which are otherwise difficult management problems. We believe sustained LC is another measure that should be used to judge the utility of more intensive therapies such as this.

Although the *post hoc* comparison with our previous study is open to patient selection bias, a comparison of patient and study characteristics showed that most known prognostic confounders favoured the earlier gemcitabine cohort. Therefore, the comparison would be expected to underestimate any advantage of the current study treatment; and moreover, any improvement with the current study treatment would be more indicative of a true benefit. Despite this, comparison of LC, PFS, and OS, all showed potential benefit over the gemcitabine study, suggesting a possible improvement from the addition of oxaliplatin.

This is supported by comparison with other previously reported studies of inoperable LAPC, in which the longest reported OS was 13.5 months, less than our median 15.7 months. In particular, a previous report using this regimen with 2 months of induction, but no consolidation, showed encouraging but slightly less disease control, suggesting, and as stated by the authors, that more prolonged systemic therapy may be required (Moureau-Zabotto *et al*, 2008). Our data has since encouraged us to join the GERCOR randomised LAP 07 study of chemotherapy alone *vs* chemotherapy followed by radiation, which should be the definitive test of how much the addition of radiation contributes to the overall outcome.

The most common reasons for early discontinuation of treatment were disease progression (nine patients) and treatment toxicity (eight patients). These apparently modest toxicity findings, none of which led to sustained morbidity, were matched by the patient-reported global QOL scores. Global scores remained stable for most patients across the treatment programme, but we did note a trend of lessening of QOL with time. Quality of life did return to baseline, which is consistent with the clinical experience of the authors (face validity), and patterns of objective toxicity. Hence, from both the objective and QOL perspective, this intensive treatment regimen appears both tolerable and safe. A limitation to this conclusion, as with all QOL studies, is that we can only report for those patients who return forms. As the majority of those not returning the forms are likely to have had disease progression, we can say that those who complete the therapy do not have an impaired QOL from the treatment.

Although outcomes appeared better than in our previous trial, which may reflect additional benefits of this regimen, we acknowledge they could still be due to chance (wide CIs) or differences in patient selection and other care.

The exploratory analyses suggested that CA19-9 levels 6 weeks after treatment began may correlate with PFS, a finding that merits investigation in a larger study, as it could be an early indicator of treatment efficacy, allowing investigators to identify patients most likely to benefit and those who may require (or be spared) more intensive treatment or simply have a worse prognosis. A higher RR correlated with a 50% reduction in CA19-9, suggesting that CA19-9 could be used to easily and quickly confirm treatment response. This is supported by data from systemic chemotherapy studies in metastatic disease (Wong *et al*, 2008; Duffy *et al*, 2010). Taken together, these results may indicate a role for CA19-9 in treatment decisions.

Recent identification of regimens with activity against metastatic disease generally suggests that even greater gains in prolonging disease control will be possible in LAPC from newer combinations and extended treatment (Conroy *et al*, 2011).

Given that those whose treatment fails early are unlikely to benefit from elective pancreatic radiation treatment, there has been a recent trend to increase the period of chemotherapy before definitive radiation (Huguet *et al*, 2007). This will identify patients with a higher risk of early systemic spread, sparing them from intensive local treatment. Our findings, that most patients complete their first consolidation treatment, but then the percentage completion rate rapidly declines, supports this. On the other hand, initiatives to improve systemic control are still needed. An area needing exploration is the addition of targeted agents (despite recent negative trials (Van Cutsem *et al*, 2009; Kindler *et al*, 2010; Philip *et al*, 2010)) for their influence on both systemic and LC, as well as interaction with radiotherapy. Progress will only occur if clinicians and patients participate in further clinical trials.

In conclusion, the intensification of induction and consolidation chemotherapy with oxaliplatin is shown to be feasible, safe, and the toxicity manageable. The better OS and LC observed, compared with the outcome of our immediately prior CRT programme, warrants further evaluation in a phase III study. The 6-week response characteristics of CA19-9 appear prognostic and also warrant further evaluation.

## Figures and Tables

**Table 1:** Baseline demographic characteristics

**Characteristic**	***N*=47**
*Age (years)*	
Age (mean±stable disease)	62.0±9.2
Age (range)	44–81
	
*Sex*	
Male (%)	31 (66.0)
Female (%)	16 (34.0)
	
*ECOG performance status*	
0 (%)	26 (55.3)
1 (%)	19 (40.4)
2 (%)	2 (4.3)
	
*Primary site*	
Head of pancreas (%)	36 (76.6)
Body of pancreas (%)	11 (23.4)
	
*T stage*	
T1 (%)	3 (6.4)
T2 (%)	15 (31.9)
T3 (%)	8 (17.0)
T4 (%)	22 (44.7)
	
*N stage*	
N0 (%)	21 (44.7)
N1 (%)	26 (55.3)

**Table 2:** Haematological and non-haematological adverse events (NCI CTCAE version 3.0)

***n*=47 patients**	**All grades (%)**	**Grade 3 (%)**	**Grade 4 (%)**
Infection without neutropenia	4 (8.6)	2 (4.3)	0 (0.0)
Anorexia	28 (59.6)	1 (2.1)	0 (0.0)
Nausea	42 (89.4)	4 (8.5)	0 (0.0)
Vomiting	23 (48.9)	2 (4.3)	0 (0.0)
Diarrhoea	25 (53.2)	2 (4.3)	0 (0.0)
Stomatitis/mucositis	9 (19.1)	1 (2.1)	0 (0.0)
Fatigue	39 (83.0)	4 (8.5)	0 (0.0)
Anaemia	29 (61.7)	5 (10.6)	0 (0.0)
Neutropenia without infection	10 (21.3)	2 (4.3)	0 (0.0)
Thrombocytopenia	26 (55.3)	1 (2.1)	1 (2.1)
Liver function (*γ*GT)	31 (66.0)	11 (23.4)	3 (6.4)
Late radiation toxicity (*n*=45 patients)	9 (20.0)	1 (2.2)	1 (2.2)

Abbreviation: NCI CTCAE=NCI Common Terminology Criteria for Adverse Events.

**Table 3:** Response rate

***n*=46 evaluable patients** a	**Number**	**% (95% CI)**
Confirmed CR	0	0.0 (0–8)
Confirmed PR	16	34.8 (23–49)
SD	25	54.3 (40–68)
PD	5	10.9 (5–23)
Clinical benefit (CR+PR+SD)	41	89.1 (77–95)

Abbreviations: CI=confidence interval; CR=complete response; PD=progressive disease; PR=partial response; SD=stable disease.

a One patient was not evaluable for response rate.

**Table 4:** Comparison with previous study characteristics

**Component**	**Confounder**	**Gem**	**GemOx**	**Favours**
Inclusion criteria	Liver function tests	No limit	<3 × ULN or stented	GemOx
	Operability	Inoperable only	Included patients strongly declining surgery	Gem
				
Baseline characteristics	Male (%)	37	67	Unknown
	ECOG performance status (0–1; %)	93	94	Neither
	T4 (%)	20	46	Gem
	N0 (%)	61	45	Gem
	Age (minimum)	30	44	Gem
				
Treatment	Schedule	Gem d1 d8 d15	GemOx d1 d16	N/A
				
Assessment	Tumour assessment	WHO	RECIST	Unknown
	CT scans during Rx	>4 weeks apart	Weeks 4, 14, 26	Unknown

Abbreviations: CT=computed tomography; N/A=not applicable; RECIST=Response Evaluation Criteria in Solid Tumours.

**Table 5:** Comparison with previous study results

	**Gem (*n*=41; 95% CI)**	**GemOx (*n*=47; 95% CI)**
Clinical benefit (CR+PR+SD; %)	48.3 (31–66)^a^	89.1 (77–95)^b^
Median sustained LC (months)	11.9 (8.9–17.9)	15.8 (10.5–17.9)
Median progression free survival (PFS) (months)	7.1 (6.3–9.2)	11.0 (8.4–13.0)
Median overall survival (OS) (months)	11.7 (9.7–13.7)	15.7 (13.1–18.3)

Abbreviations: CI=confidence interval; CR=complete response; LC=local control; PD=progressive disease; PR=partial response; SD=stable disease.

a *n*=29 evaluable patients.

b *n*=46 evaluable patients.
