# Supplementary material for: The GOFURTGO Study: AGITG Phase II Study of fixed dose rate gemcitabine–oxaliplatin integrated with concomitant 5FU and 3-D conformal radiotherapy for the treatment of localised pancreatic cancer
Source: Br J Cancer. 2011 Dec 1;106(1):61–9. doi: 10.1038/bjc.2011.526 (PMC3251866; doi:10.1038/bjc.2011.526)
Supplement: Supplementary Information [file bjc2011526x1.doc]

**GOFURTGO Online Supplement**

**RADIATION TECHNIQUE**

Patients were immobilised using a reliable set up apparatus, and required to be supine with arms over head during simulation and treatment.

Planning CT scan thickness could be no thicker than 5 mm and at no greater than 5-mm intervals to minimize extent of volume averaging and provide good tumor and normal tissue imaging. Intravenous contrast was advised to assist definition of the major abdominal arterial structures, and oral contrast was recommended to visualize the duodenal loop. Volume extension to incorporate “at risk” nodal sites was discouraged because node prophylaxis in this setting has not yet been shown to be beneficial, the main site of failure continues to be local mass site and because radiotherapy-induced toxicity increases with treatment volume size.

**Technique**

The PTV was extended in the superior–inferior plane to allow for respiratory movement. Normal tissue tolerance dose constraints for kidney, liver, and spinal cord were as defined by Emami et al, 1991. The dose was prescribed in accordance with the International Commission on Radiation Units and Measurements ICRU 50 Report (ICRU, 1993) and was 54 Gy in 30 fractions of 1.8 Gy. Three-dimensional conformal techniques, coplanar or noncoplanar, were acceptable. Intensity-modulated radiation therapy techniques were not employed in Australian radiotherapy practice at the time of the study. Quality assurance for radiotherapy was as described previously (Spry et al, 2008).

References:

Emami B, Lyman J, Brown A, Coia L, Goitein M, Munzenrider JE, Shank B, Solin LJ, Wesson M (1991) Tolerance of normal tissue to therapeutic irradiation. Int J Radiat Oncol Biol Phys 21: 109 –122

ICRU (1993) Report No. 50, Prescribing, Recording, and Reporting Photon Beam Therapy. Bethesda, MD: International Commission on Radiation Units and Measurements

Spry N, Harvey J, Macleod C, Borg M, Ngan SY, Millar JL, Graham P, Zissiadis Y, Kneebone A, Carroll S, Davies T, Reece WH, Iacopetta B, Goldstein D (2008) 3D radiotherapy can be safely combined with sandwich systemic gemcitabine chemotherapy in the management of pancreatic cancer: factors influencing outcome. Int J Radiat Oncol Biol Phys 70(5): 1438-46, doi:10.1016/j.ijrobp.2007.08.070

**OUTCOME DEFINITIONS**

**Efficacy Criteria for Tumor Response**

- **Complete response (CR):** Complete disappearance of all measurable and evaluable disease. No new lesions. No disease-related symptoms. No evidence of non-evaluable disease, including normalization of markers and other abnormal lab values. All measurable, evaluable, and non-evaluable lesions and sites must be assessed using the same technique as baseline.
- **Partial response (PR):** Equal to or greater than a 30% decrease relative to baseline in the sum of LD of all measurable disease. No progression of any lesion. No new lesions. All measurable and evaluable lesions and sites must be assessed using the same techniques as used at baseline.
- **Stable disease (SD):** Does not qualify for CR, PR, or progressive disease. All measurable and evaluable lesions and sites must be assessed using the same techniques as baseline.
- **Progressive disease (PD):** Equal to or greater than a 20% increase in the sum of LD of all measured lesions (measurable disease) relative to smallest sum observed (or relative to baseline if no decrease) using the same techniques as used at baseline, OR reappearance of any lesion which had disappeared, OR appearance of any new lesion/site, OR failure to return for evaluation due to death or deteriorating condition (unless clearly unrelated to this cancer). Worsening of existing non-evaluable disease does not constitute progression.

Exception: Lesions that appear to increase in size due to presence of necrotic tissue will not be considered to have progressed.

- **Unknown**: Progression has not been documented and one or more measurable or evaluable sites have not been assessed.

**Best objective tumour response** is determined as follows:

‐ Two objective status determinations of CR before disease progression are required for a best response of CR.

‐ Two determinations of PR before disease progression are required for a best response of PR.

‐ Two determinations of SD before disease progression are required for a best response of SD.

‐ Patients with an objective status of PD on or before the second evaluation will have a best response of PD.

‐ Best Response is considered unknown if the patient only had one assessment.

**RECIST Response Rate** is defined as the percentage of patients with Best response CR + PR / total number of evaluable patients.

**RECIST Response Rate (excluding early withdrawals)** is defined as per RECIST Response Rate, but will be computed excluding patients who withdraw early (i.e. within 30 days of registration) for any reason.

**Overall survival** (months) is defined as the number of days between date of death (or date of last follow‐up) and date of registration / 30.4.

**Progression Free Survival** is defined as time (months) from registration to documented evidence of disease progression, the occurrence of new disease or death from any cause.

**Local control** is defined as a patient not having had locoregional progression. Patients who progress whilst maintaining local control are censored at the date of their last CT scan.

**Time to local control** (months) is defined as the number of days between date of local control (or date last known not to have local control) and the date of registration / 30.4.

**Exploratory analysis of baseline factors predicting survival**

The following variables were prospectively selected for exploratory analysis of predictive factors:

- ECOG disease status
- Age
- White blood cell count
- Tumour size
- CA19.9 tumour biomarker
- Tumour stage
- Lymph-node stage

Kaplan-Meier survival estimates were used to summarise the groups. Unadjusted proportional hazards Coxregression models will be used to determine the size and 95% confidence intervals of all treatment effects. The Harrell-Lee test was used to test the proportional hazards assumption, and if the hazards were demonstrated to be

Non-proportional, the Prentice-Peto adjusted log-rank statistic is reported.

Categorical grouping of continuous variables was made considering clinically intuitive cut-points to enable simple interpretation. These variables will also be investigated as continuous variables in the models to check for consistency. Variables were to be considered potential univariate predictors if they had a p-value < 0.05.

**Online Table 1. Univariate results – progression free survival**

| **Factor (Group, Reference)** | **HR, (95% CI), p-value** | **PH test** |
| --- | --- | --- |
| ECOG status (1/2 v 0) | 1.71 (0.92, 3.17), p=0.09 | p=0.85 |
| White cell count (continuous) | 1.03 (0.92, 1.16), p=0.55 | p=0.46 |
| Age group (≥60 v <60) | 0.65 (0.35, 1.21), p=0.18 | p=0.76 |
| *Age (continuous, 10years) | 0.76 (0.52, 1.10), p=0.15 | p=0.20 |
| Tumour stage (T3/T4 v T2/T1) | 1.05 (0.57, 1.95), p=0.87 | p=0.66 |
| Nodal stage (N1 v N0) | 0.77 (0.41, 1.48), p=0.45 | p=0.63 |
| CA19.9 group (100-1000 v <100) | 0.91 (0.45, 1.84), p=0.79 | p=0.23 |
| CA19.9 group (≥1000 v <100) | 1.72 (0.74, 4.00), p=0.21 | p=0.75 |
| *CA19.9 (continuous, log_10_) | 1.26 (0.93, 1.71), p=0.13 | p=0.74 |

**Online Table 2. Univariate results – overall survival**

| **Factor (Group, Reference)** | **HR, (95% CI), p-value** | **PH test** |
| --- | --- | --- |
| ECOG status (1/2 v 0) | 1.41 (0.78, 2.58), p=0.26 | p=0.91 |
| White cell count (continuous) | 0.93 (0.83, 1.04), p=0.19 | p=0.51 |
| Age group (≥60 v <60) | 0.66 (0.36, 1.22), p=0.19 | p=0.62 |
| *Age (continuous, 10years) | 0.88 (0.61, 1.22), p=0.45 | p=0.84 |
| Tumour stage (T3/T4 v T2/T1) | 1.12 (0.63, 2.16), p=0.61 | p=0.11 |
| Nodal stage (N1 v N0) | 0.90 (0.48, 1.71), p=0.75 | p=0.69 |
| CA19.9 group (100-1000 v <100) | 1.01 (0.49, 2.03), p=0.99 | p=0.82 |
| CA19.9 group (≥1000 v <100) | 2.33 (0.98, 5.51), p=0.053 | p=0.60 |
| *CA19.9 (continuous, log10) | 1.44 (1.02, 2.04), p=0.037 | p=0.88 |

**Exploratory comparison of CA19.9 and RECIST**

**Online Table 3. Association with RECIST and CA19.9 reduction**

|  | | RECIST Response | | | | | |
| --- | --- | --- | --- | --- | --- | --- | --- |
|  | | Yes | | No | | Total | |
|  | | Count | % | Count | % | Count | % |
| CA19-9 Reduced | Reduced (>=50%) | 13 | 50% | 13 | 50% | 26 | 100.0% |
|  | Not Reduced (>=50%) | 3 | 15% | 17 | 85% | 20 | 100.0% |
|  | Total | 16 | 35% | 30 | 65% | 46 | 100.0% |

Despite the small sample size there is indication of a weak relationship of increased odds of response if CA19.9 is reduced (OR=5.2, 95%CI (1.3, 24), p‐value=0.019).

**Landmark analysis -** Patients classified as having either increasing or decreasing CA19.9 values at 6 weeks after registration. PFS / OS are assessed from 6 weeks onwards (for those patients who are still at risk at that time).

**Online Figure 1. PFS after 6 week landmark**

**Online Table 4. Time to progression using CA19.9 status at 6 weeks**

|  | | Progression (48 patients) | | | | Survival (48 patients) | | | |
| --- | --- | --- | --- | --- | --- | --- | --- | --- | --- |
|  |  | HR | 95% CI for HR | | P-Value | HR | 95% CI for HR | | P-Value |
|  |  |  | Lower | Upper |  |  | Lower | Upper |  |
| CA 19.9 after 6 weeks | Unknown vs. Increasing | 0.34 | 0.14 | 0.84 | 0.02 | 0.74 | 0.31 | 1.72 | 0.49 |
|  | Reducing vs. Increasing | 0.36 | 0.18 | 0.73 | 0.005 | 0.72 | 0.36 | 1.44 | 0.35 |
